# A Series of Clinical Cases

**Published:** 1897-08

**Authors:** Vida A. Latham

**Affiliations:** Chicago, Ills.


### A Series of Clinical Cases.

BY VIDA A. LATHAM, M.D., D.D.S., CHICAGO, ILLS.

Abstract of paper read at Dental Section Amer. Med. Asso., June, 1897.

Every practitioner of dentistry, at some time or other, sees cases of this nature, perhaps meeting the disease in its earliest form. If he is able to recognize it and sound a note of warning, even though he may prefer to refer the case to a specialist, he has done a great favor to the patient, and has earned his gratitude.

### MYELOID SARCOMA OF THE JAW.

Case 1. Not long since I saw a case which had been sent to a dentist for advice regarding a right superior third molar that was troublesome. The patient was told to have the tooth removed, "as the wisdom teeth were of no value," and this was accordingly done. But there was no advice or suggestion of anything suspicious about the thickening of the gum around the outer side of the tooth. After a few months the patient became a medical student, and, as the socket showed some irritability, a Junior teacher and dentist in the University school was consulted. He pronounced the growth nothing but thickened gum and it was snipped off and thrown into the cuspidor, and the place touched with a counter-irritant. In a little while it was thought to be recurring but the dentist said, "No, it was all right," and as he held a place in Operative Dentistry and was Quiz Master in the subject, the matter was left on his advice. Only a few months later the student transferred to another school and happened to hear a lecture on the subject, the importance of which started a feeling of uneasiness. At this time I made the acquaintance of the student, who consulted me for examination and advice. Truly, the growth was small and seemingly innocent and fibroid in character, and I asked leave to watch the case for a short time. Finding that it grew larger I advised thorough and immediate operation, as I feared a sarcomatous epulis. On account of the position of the former dentist and not wishing to seem to urge an operation on a small matter, I suggested the consultation of an older and more experienced specialist than myself. After a little persuasion the patient consented to the removal of a small piece of tissue for examination. The opposite end of the tissues from the neighborhood of the periosteal surface showed the early but rapidly-growing and malignant nature of the tissue—a typical myeloid or giant-celled sarcoma. The patient who knew something of the danger of such a growth was eager for a thorough radical operation which was performed by the consulting oral surgeon. After eighteen months there has been no recurrence. The great danger in this case lay in the fact that the growth first appeared behind and on the buccal surface of the gum near the third molar, and its course of progression would have been up the palate process along the maxillary tuberosity and posterior palatine canal, so invading the antrum and palatoglossal folds. It is generally believed that myeloid sarcomata are common in the maxillæ, though Bland Sutton says they are rare, and as a rule arise in connection with the nasal process. In the maxillæ or mandible they arise from the body of the bone.

SMALL SPINDLE-CELLED SARCOMA OF THE PALATE.

Case 2. In this case the patient, aged 33 years, had been in perfect health up to six months previous to the time he came under my observation. There appeared a thickening on the left lingual surface of the palate. At first the patient was but little concerned, thinking it only a local irritation, but as it slowly increased in size, he consulted a dentist who told him it was a chronic inflammatory growth, and possibly due to his smoking a pipe. This habit was discontinued and the swelling treated by counter-irritation which gave no relief. By this time the place was a source of annoyance as the patient could not keep his tongue from it, and he determined to have it removed by his family physician. The latter, upon examination of the growth, found that it extended from the first molar to the soft palate within half an inch of uvula, and becoming suspicious of its nature, he decided to prepare for a radical operation and gained the patient's consent to do what he deemed best. The growth was periosteal in origin and was thoroughly extirpated, the teeth being extracted on that side and the alveolar border curetted, removing all tissue that looked dangerous or even suspicious. There has been no recurrence. The differential diagnosis between palatal abscess and sarcoma shows the following points: Sarcoma springing from the periosteum of the hard palate appears as a soft, semi-elastic, more or less rapidly-growing tumor affecting the roof of the mouth. A palatal abscess originating from any of the upper teeth or burrowing backwards beneath the bone and periosteum, or in the soft tissues forming the roof of the mouth, gives rise to pain, and inflammation attends its development; it extends more rapidly, and gives an elastic feeling of fluctuation. There is the evidence of a carious tooth and in chronic cases the diagnosis can be made positively by using exploratory puncture.

SARCOMA OF ANTRUM.

Case 3. The patient, a well-built boy of 15 years, complained of constant pain in the left upper first molar, which was treated. In about three weeks his cheek began to swell, and on returning to the dentist the boy was informed that the swelling was an abscess. Free incisions were made and no pus found. As he got no better and the pain continued, the patient left his dentist going to one of the well-known advertising houses, and there was told to have the tooth extracted. This was done and after the somewhat profuse hemorrhage, which followed, the pain was better. The swelling, however, did not disappear, and I saw the case upon recommendation of the family physician. The unilateral bulging of the antrum, and upward displacement of the eye, the slight amount of pain, together with the history and previous treatment, led to the diagnosis of sarcoma of rapid growth. I gave the patient an unfavorable prognosis and urged a speedy removal of the upper jaw, which was done. Then the soft parts were laid back and the disease found to be very extensive, requiring the removal of the entire superior maxillæ and malar bones, the zygoma, and part of the temporal and masseter muscles. Hemorrhage was very severe, but was controlled by iodoform packing and compound tincture benzoin. In spite of this bold and severe operation the disease recurred and the patient died from exhaustion. The microscope showed the tumor to be a small, round-celled sarcoma.

